# Genome sequence of *Clostridium sporogenes* DSM 795^T^, an amino acid-degrading, nontoxic surrogate of neurotoxin-producing *Clostridium botulinum*

**DOI:** 10.1186/s40793-015-0016-y

**Published:** 2015-07-21

**Authors:** Anja Poehlein, Karin Riegel, Sandra M König, Andreas Leimbach, Rolf Daniel, Peter Dürre

**Affiliations:** Institute of Microbiology and Biotechnology, University of Ulm, 89069 Ulm, Germany; Genomic and Applied Microbiology & Göttingen Genomics Laboratory, Institute of Microbiology and Genetics, Georg-August University Göttingen, Göttingen, Germany

**Keywords:** C. *sporogenes*, Anaerobic, Butanol, *C. botulinum*, Gram-positive, Stickland reaction

## Abstract

*Clostridium sporogenes* DSM 795 is the type strain of the species *Clostridium sporogenes*, first described by Metchnikoff in 1908. It is a Gram-positive, rod-shaped, anaerobic bacterium isolated from human faeces and belongs to the proteolytic branch of clostridia. *C. sporogenes* attracts special interest because of its potential use in a bacterial therapy for certain cancer types.

Genome sequencing and annotation revealed several gene clusters coding for proteins involved in anaerobic degradation of amino acids, such as glycine and betaine via Stickland reaction. Genome comparison showed that *C. sporogenes* is closely related to *C. botulinum*. The genome of *C. sporogenes* DSM 795 consists of a circular chromosome of 4.1 Mb with an overall GC content of 27.81 mol% harboring 3,744 protein-coding genes, and 80 RNAs.

**Electronic supplementary material:**

The online version of this article (doi:10.1186/s40793-015-0016-y) contains supplementary material, which is available to authorized users.

## Introduction

*C. sporogenes* was isolated from human faeces [1-3], but can also be found in soil and marine or fresh water sediments [4-7]. *C. sporogenes* strain DSM 795 [8] serves as type strain for this species and as a consequence was chosen for whole genome sequencing.

Because *C. sporogenes* is closely related to *C. botulinum* group I strains, it is used as a non-toxic surrogate for this common food-borne pathogen. 16S rDNA sequencing revealed a 99.7% sequence similarity to proteolytic *C. botulinum* strains of serotypes A, B, and F [9]. In this context, the genome of *C. sporogenes* strain PA 3679 was sequenced and a draft sequence published in 2012 [10]. *C. sporogenes* can be isolated from infections, but does not play a prominent role as a pathogen. Only few clinical cases are reported, in which this species was found to participate. These cases include epynema, soft tissue abscesses, septic arthritis, or gas gangrene [11-16].

Requiring an anaerobic habitat, *C. sporogenes* is known to specifically colonize hypoxic areas of solid tumors. In 1964, Möse and co-workers demonstrated tumor colonization resulting in tumor lysis after intravenous application of *C. butyricum* M-55 in mice carrying Ehrlich carcinomas [17]. The respective strain was subsequently reclassified as *C. oncolyticum* and finally as *C. sporogenes*ATCC 13732. They also demonstrated that *C. sporogenes* spores are immunologically inert by injecting them into themselves [18]. As an excellent tumor colonizer, *C. sporogenes* bears promising therapeutic potential for cancer therapy [19]. With CFU numbers up to 2 × 10^8^ per gram of tumor tissue, *C. sporogenes* outperforms saccharolytic clostridia such as *C. beijerinckii* and *C. acetobutylicum* by far, as the latter reach only CFU numbers of 10^5^-10^6^ per gram of tumor tissue [20-22].

Restricted to the inner core of the tumor, clostridia cannot lyse the well-oxygenated outer rim of tumor cells, which remains viable and unaffected. Therefore, treatment with clostridial spores alone is not sufficient for complete tumor eradication. Several attempts have been made to genetically modify *C. sporogenes* for production of therapeutic proteins or pro-drug converting enzymes. The latter catalyze the conversion of innocuous pro-drug molecules into their active, cytotoxic form. This reaction takes place directly in the tumor allowing systemic application of higher drug concentrations and reduction of side effects [20]. Among others, cytosine deaminase and nitroreductase were used for this purpose. A recombinant *C. sporogenes*DSM 795 mutant expressing cytosine deaminase induced growth delay of tumors in a mouse model after application of spores and the pro-drug 5-fluorouracil [20]. Also, *C. sporogenes* mutants heterologously expressing nitroreductase exhibited a significant antitumor efficacy in different *in vivo* tumor models [23-25].

## Organism information

### Classification and features

*C. sporogenes* has been subject of extensive studies since the 1930s. Characteristic features of *C. sporogenes*DSM 795 are listed in Table 1.

**Table 1:** **Classification and general features of** ***Clostridium sporogenes*** **DSM 795** ^**T**^

**MIGS ID**	**Property**	**Term**	**Evidence code** ^**a**^
	Classification	Domain *Bacteria*	[26]
		Phylum *Firmicutes*	[27-29]
		Class *Clostridia*	[30,31]
		Order *Clostridiales*	[32,33]
		Family *Clostridiaceae*	[32,34]
		Genus *Clostridium*	[32,35,36]
		Species *Clostridium sporogenes*	[8,32,37,38]
		Type strain DSM 795 ^T^	[8]
	Gram stain	positive	IDA
	Cell shape	rod-shaped	IDA
	Motility	motile	[39]
	Sporulation	sporulating	IDA
	Temperature range	mesophilic, 25–45°C	[39]
	Optimum temperature	30-40°C	IDA, [39]
	pH range; Optimum	5.7-8.5; 7	[39], IDA
	Carbon source	amino acids	[40-43]
MIGS-6	Habitat	human and animal gut, soil, marine and fresh water sediments	[4-7,44,45]
MIGS-6.3	Salinity	growth in 2YT medium	IDA
MIGS-22	Oxygen requirement	anaerobic	IDA
MIGS-15	Biotic relationship	free living	IDA
MIGS-14	Pathogenicity	low	[39]
MIGS-4	Geographic location	Not reported	
MIGS-5	Sample collection	Not reported	
MIGS-4.1	Latitude	Not reported	
MIGS-4.2	Longitude	Not reproted	
MIGS-4.4	Altitude	Not reproted	

^a^Evidence codes - IDA: Inferred from Direct Assay. Evidence codes from the Gene Ontology project [46].

*C. sporogenes* belongs to the proteolytic branch of clostridia capable of amino acid fermentation. No carbohydrates are required for growth, although addition can have a stimulating effect [47]. Amino acids are degraded via the Stickland reaction for energy conservation [40-43]. Required media composition and further nutritional demands have already been elucidated [48-55]. Growth can be obtained anaerobically in complex medium, but also several minimal media supplemented with amino acids are described [52,53].

**Figure 1:**
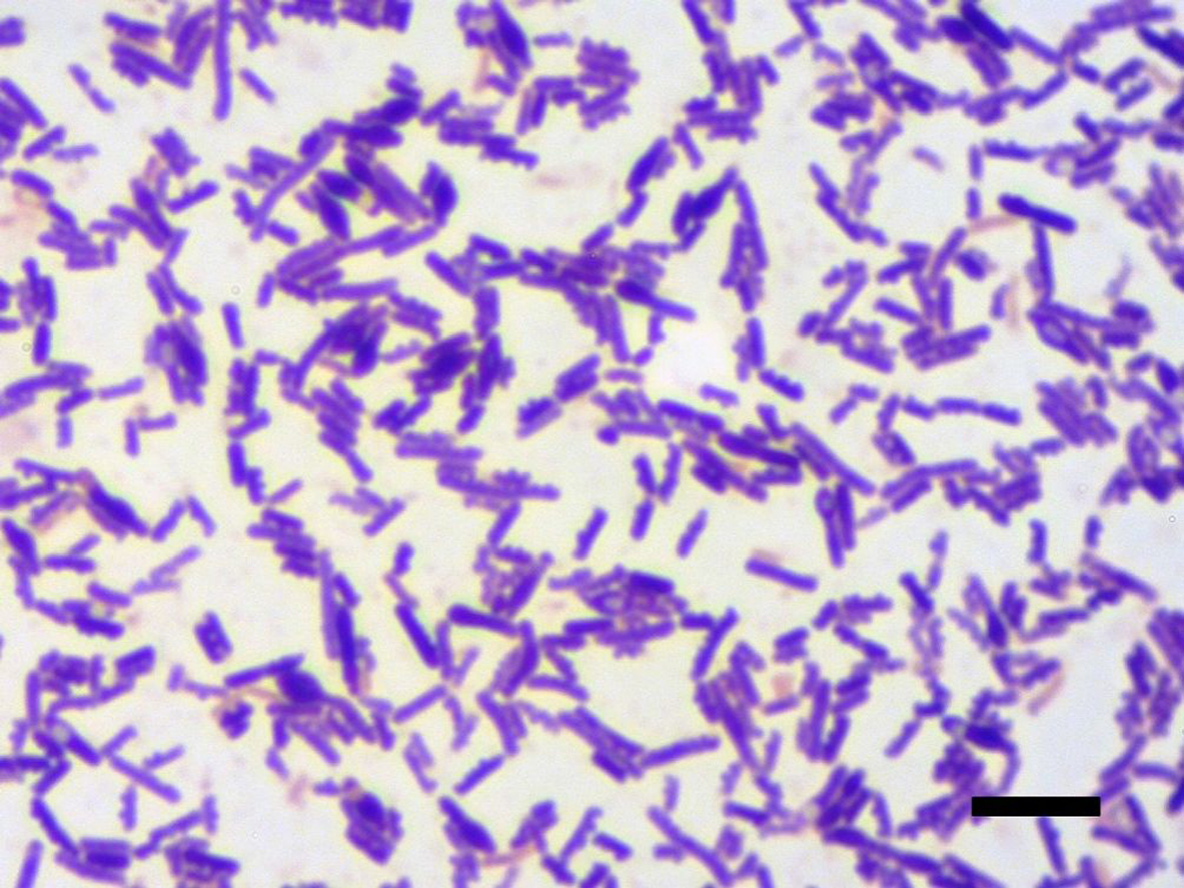
Gram staining of Gram-positive *C. sporogenes* DSM 795. Scale bar represents 5 μm.

The Gram-positive nature of *C. sporogenes* was confirmed by Gram staining (Figure 1). Cell size can vary between 0.3-1.4 × 1.3-16.0 μm [39].

**Figure 2:**
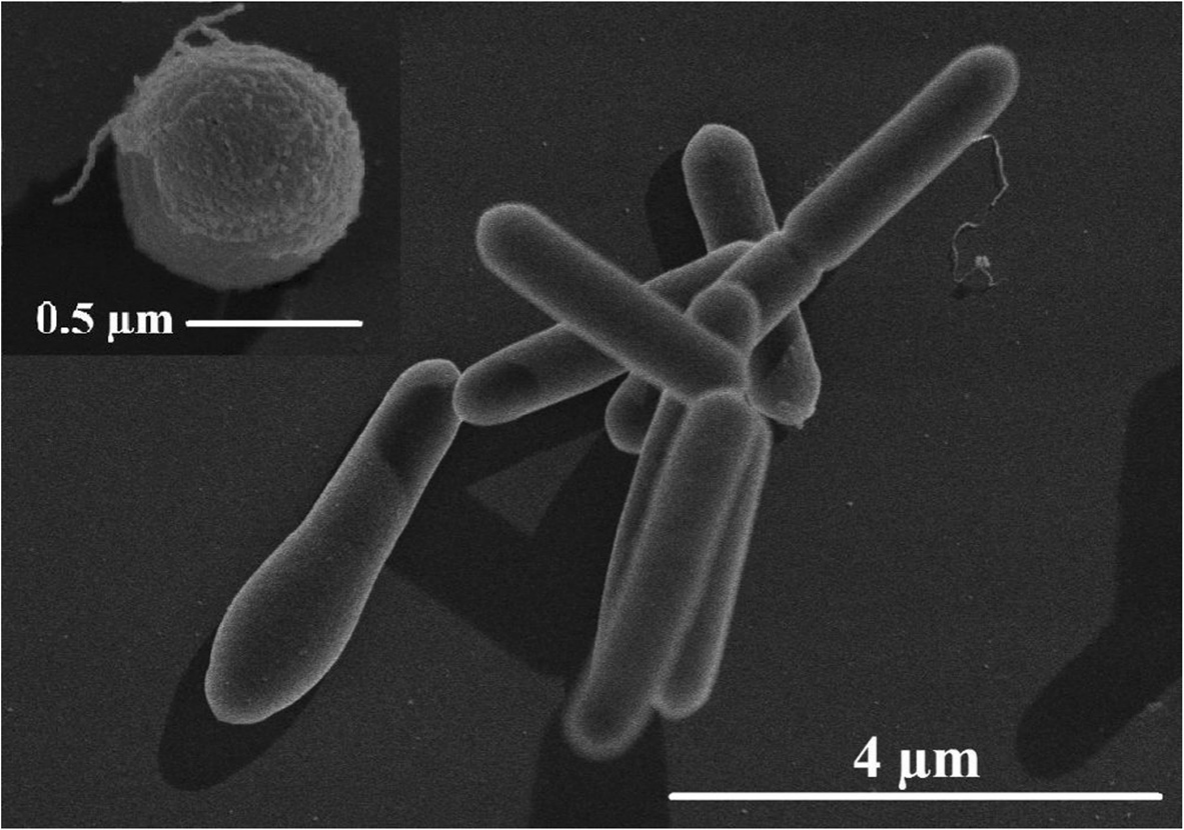
Scanning electron microscopy image of *C. sporogenes* DSM 795.

A scanning electron microscopy image of *C. sporogenes*DSM 795 cell culture is shown in Figure 2. Several cell stages are depicted: vegetative dividing and sporulating cells and a mature spore (upper left part of the image).

**Figure 3:**
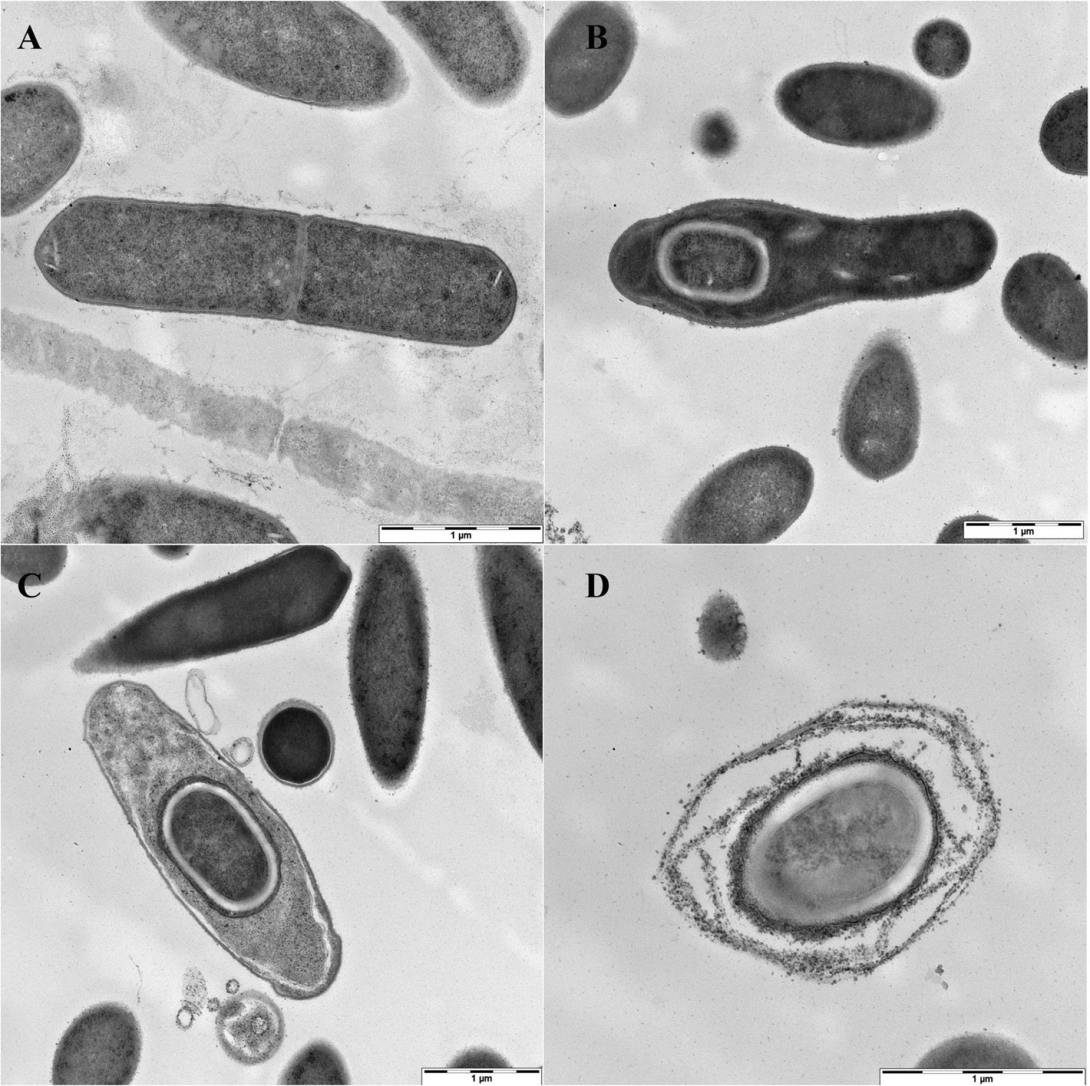
Transmission electron microscopy image of *C. sporogenes* DSM 795; **A**: dividing cell; **B**, **C**: sporulating cells; **D**: spore; scale bars represent 1 μm.

Transmission electron microscopy images (Figure 3) reveal membrane organizations and cell compartments of a dividing cell (Figure 3A), sporulating cells (Figure 3B and C), and a spore (Figure 3D).

*C. sporogenes* is considered as the non-toxic surrogate of neurotoxin producer *Clostridium botulinum*. Additional file 1: Table S1 provides an overview of all C. *botulinum* strains mentioned in this study. Generally, they are assigned to four groups (I-IV) based on their physiologic characteristics [56]. Strains belonging to group I are proteolytic [57]. They are further classified into serotypes A-F due to different types of the produced botulinum neurotoxin with several subtypes existing [56].

**Figure 4:**
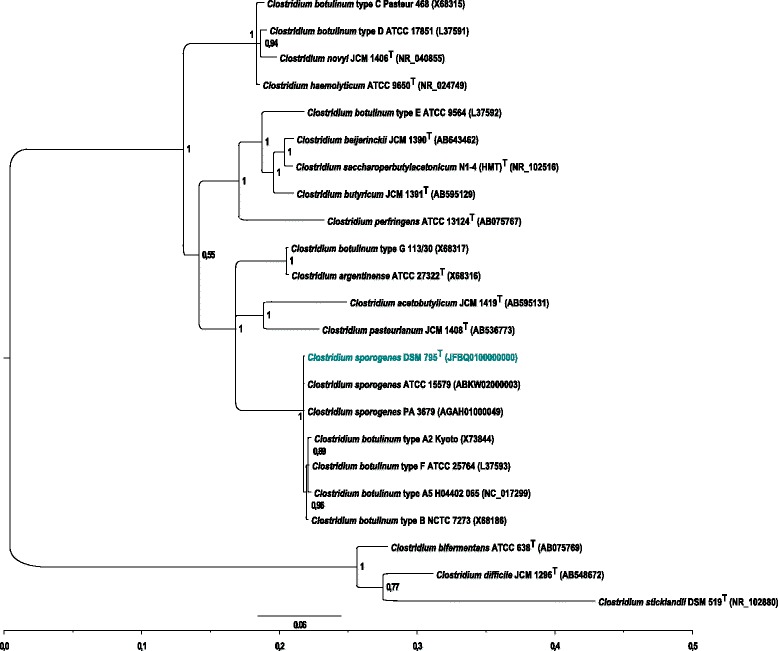
Phylogenetic tree of *C. sporogenes* DSM 795 based on 16S rDNA gene sequences. Estimation is based on Bayesian inference and MAFFT alignment.

Phylogenetic relation of *C. sporogenes*DSM 795 to *C. botulinum* strains and other clostridia was investigated by calculation of a phylogenetic tree using 16S rDNA sequences (Figure 4). *C. sporogenes*DSM 795 positions itself in close relationship to proteolytic *C. botulinum* strains of types A, B, and F, confirming previous studies [9]. Clostridial 16S rRNA reference sequences were retrieved from GenBank (NCBI database). At first, these sequences were aligned with MAFFT version 7 using default settings except for “globalpair” in fast Fourier transform [58]. Then, based on the multiple sequence alignment, a phylogenetic tree was inferred with the program MrBayes 3.1.2 [59] using the default settings.

*C. sporogenes*DSM 795 exhibits β-hemolysis on sheep and human erythrocytes (data not shown) due to production of clostridiolysin S [60]. Further enzymes produced are desoxyribonuclease, thiaminase, chitinase, kininase, L-methioninase, hyaluronate lyase, and superoxide dismutase [39].

In general, *C. sporogenes* is resistant to streptomycin, neomycin, kanamycin, tobramycin, and amikamycin and susceptible to penicillin G, metronidazole, tinidazole, chloramphenicol, tetracycline, and doxycycline [39]. *C. sporogenes* strain DSM 795 is additionally susceptible to thiamphenicol and erythromycin or clarithromycin in working concentrations of 15 μg/ml and 2.5 μg/ml, respectively.

## Genome sequencing information

### Genome project history

*C. sporogenes*DSM 795 was chosen for whole genome sequencing as it is the type strain of this species. Furthermore, it attracts special interest because of its potential use in tumor therapy and it is known as nontoxic surrogate of the food-borne and neurotoxin-producing pathogen *C. botulinum*. The sequencing of *C. sporogenes*DSM 795 genomic DNA delivered a high-quality draft genome sequence comprising 1 scaffold and 16 contigs. The sequence is deposited in GenBank database under the accession JFBQ00000000. A summary of the project information is listed in Table 2.

**Table 2:** **Project information**

**MIGS ID**	**Property**	**Term**
MIGS-31	Finishing quality	Improved high-quality draft
MIGS-28	Libraries used	Two genomic libraries: 454 pyrosequencing shotgun library, Illumina paired-end library (1 kb insert size)
MIGS-29	Sequencing platforms	454 GS FLX Titanium, Illumina GAII and MiSeq
MIGS-31.2	Fold coverage	11.53 × 454, 51.25 × Illumina
MIGS-30	Assemblers	Newbler 2.8, MIRA 3.4
MIGS-32	Gene calling method	YACOP, Glimmer
	Locus Tag	CSPO
	Genbank ID	JFBQ00000000
	GenBank Date of Release	2014-05-06
	GOLD ID	Gi0006347
	BIOPROJCT	239205
MIGS-13	Source material identifier	DSM 795
	Project relevance	medical, butanol formation, amino acid degradation

### Growth conditions and genomic DNA preparation

*C. sporogenes*DSM 795 was cultivated in anaerobic 2YT medium containing 3% (w/v) tryptone, 2% (w/v) yeast extract, and 8.7 mM sodium thioglycolate ([61], mod.).

For genomic DNA preparation via the hot phenol method, an overnight culture incubated at 37°C was used. The procedure was carried out as described previously [62]. Redistilled and in TE buffer equilibrated phenol (pH 7.5-8) was used for the extraction.

Electron microscopic images were taken from an overnight and a sporulating culture (10 d, 30 °C) of *C. sporogenes*. SEM and TEM cell samples were washed 3 times with PBS and fixed with 1 vol 5% (v/v) glutaraldehyde in 0.2 M phosphate buffer pH 7.3 containing 2% (w/v) sucrose. Further treatment and visualization were conducted by the Central Facility for Electron Microscopy, University of Ulm.

### Genome sequencing and assembly

Whole-genome sequencing of *C. sporogenes* was performed with a combined approach using the 454 GS-FLX Titanium XL system (Titanium GS70 chemistry, Roche Life Science, Mannheim, Germany), the Genome Analyzer II, and the MiSeq (Illumina, San Diego, CA). Shotgun libraries were prepared according to the manufacturer’s protocols, resulting in 126,343 reads for 454 shotgun sequencing (11.53 × coverage) and 1,445,024 112-bp and 5,654,920 150-bp paired-end Illumina reads (263.72 × coverage). For the initial hybrid *de novo* assembly with MIRA 3.4 [63] and Newbler 2.8 (Roche Life Science, Mannheim, Germany), we used all of the 454 shotgun reads, 1,445,024 112-bp and 554,976 150-bp paired-end Illumina reads. The final assembly was composed of 298 contigs with an average coverage of 62.78. For scaffolding we used the Move Contigs tool of the Mauve Genome Alignment Software [64]. Additionally, contigs that could not be ordered with Mauve were examined via Gene Ortholog Neighborhoods based on bidirectional best hits implemented at the IMG-ER (Integrated Microbial Genomes-Expert Review) system [65,66]. For contig ordering tasks, the genomes of *C. sporogenes*ATCC 15579 (ABKW00000000), *C. botulinum*ATCC 3502 (AM412317, AM412318), and *C. botulinum* BoNT/B1 Okra (CP000939) were used as references. Sequence gaps were closed in the Gap4 (v.4.11) software of the Staden Package [67] by PCR-based techniques and primer walking with conventional Sanger sequencing, using BigDye 3.0 chemistry on an ABI3730XL capillary sequencer (Applied Biosystems, Life Technologies GmbH, Darmstadt, Germany). The resulting draft genome is composed of 16 contigs in 1 scaffold.

### Genome annotation

The software tools YACOP and Glimmer [68] were used for automatic gene prediction, while identification of rRNA and tRNA genes was performed with RNAmmer and tRNAscan, respectively [69,70]. Automatic annotation was carried out with the IMG-ER (Integrated Microbial Genomes-Expert Review) system [65,66], but annotation was afterwards manually curated by employing BLASTP and the Swiss-Prot, TrEMBL, and InterPro databases [71].

## Genome properties

The draft genome of *C. sporogenes*DSM 795 consists of one scaffold containing 16 contigs representing one circular chromosome with a size of 4.1 Mb and with an overall GC content of 27.81 mol%. 3,832 genes are encoded, from which 3,744 were putative protein coding, 8 were pseudo and 80 RNAs (10 rRNA and 70 tRNA genes). 77.51% of encoding genes could be assigned to a putative function while the remaining 843 genes were annotated as hypothetical proteins. The genome harbors 6 different selenocysteine-containing proteins, even SelD, the selenide water dikinase (CSPO_9c05010), necessary for incorporation of selenocysteine into proteins, contains one selenocysteine. 4 of the remaining gaps represent rRNA gene clusters and there are some indications in the draft genome that at least 4 of these clusters are double, triple, or even fivefold clusters. Statistics and genome properties are listed in Table 3.

**Table 3:** **Genome statistics**

**Attribute**	**Value**	**% of Total**
Genome size (bp)	4,106,655	100.00%
DNA coding (bp)	3,416,102	83.18%
DNA G + C (bp)	1,142,131	27.81%
Number of scaffolds	16	
Total genes	3,832	100.00%
Protein coding genes	3,752	97.91%
RNA genes	80	2.09%
Pseudo genes	8^a^	
Genes in internal clusters	2,960	76.77%
Genes with function prediction	2,942	77.51%
Genes assigned to COGs	2,456	64.09%
Genes with Pfam domains	3,139	81.92%
Genes with signal peptides	176	4.59%
Genes with transmembrane helices	1,059	27.64%
CRISPR repeats	0	

^a^Pseudo genes may also be counted as protein coding or RNA genes, so are not additive under total gene count.

## Insights from the genome sequence

Of all protein coding genes 2,456 (64.09%) could be assigned to at least one COG category [72]; Table 4 shows the distribution into these functional groups. The two most abundant categories were “general function prediction” and “transcription”, to which 11.75% and 10.55%, respectively, could be assigned to, followed by “amino acid transport and metabolism”, “function unknown”, “signal transduction and mechanisms”, and “energy production and conversion” with 9.42%, 8.51%, 7.05% and 6.07%, respectively, of all protein coding genes.

**Table 4:** **Number of genes associated with the general COG functional categories**

**Code**	**Value**	**% Age**	**Description**
J	167	6.07	Translation, ribosomal structure and biogenesis
A	n. a.	n. a.	RNA processing and modification
K	290	10.55	Transcription
L	110	4.00	Replication, recombination and repair
B	n. a.	n. a.	Chromatin structure and dynamics
D	28	1.02	Cell cycle control, cell division, chromosome partitioning
V	99	3.60	Defense mechanisms
T	194	7.05	Signal transduction mechanisms
M	130	4.73	Cell wall/membrane biogenesis
N	72	2.62	Cell motility
U	34	1.24	Intracellular trafficking and secretion, and vesicular transport
O	81	2.95	Posttranslational modification, protein turnover, chaperones
C	156	5.67	Energy production and conversion
G	131	4.76	Carbohydrate transport and metabolism
E	259	9.42	Amino acid transport and metabolism
F	78	2.84	Nucleotide transport and metabolism
H	124	4.51	Coenzyme transport and metabolism
I	49	1.78	Lipid transport and metabolism
P	164	5.96	Inorganic ion transport and metabolism
Q	27	0.98	Secondary metabolites biosynthesis, transport and catabolism
R	323	11.75	General function prediction only
S	234	8.51	Function unknown
-	1,376	35.91	Not in COGs

**Figure 5:**
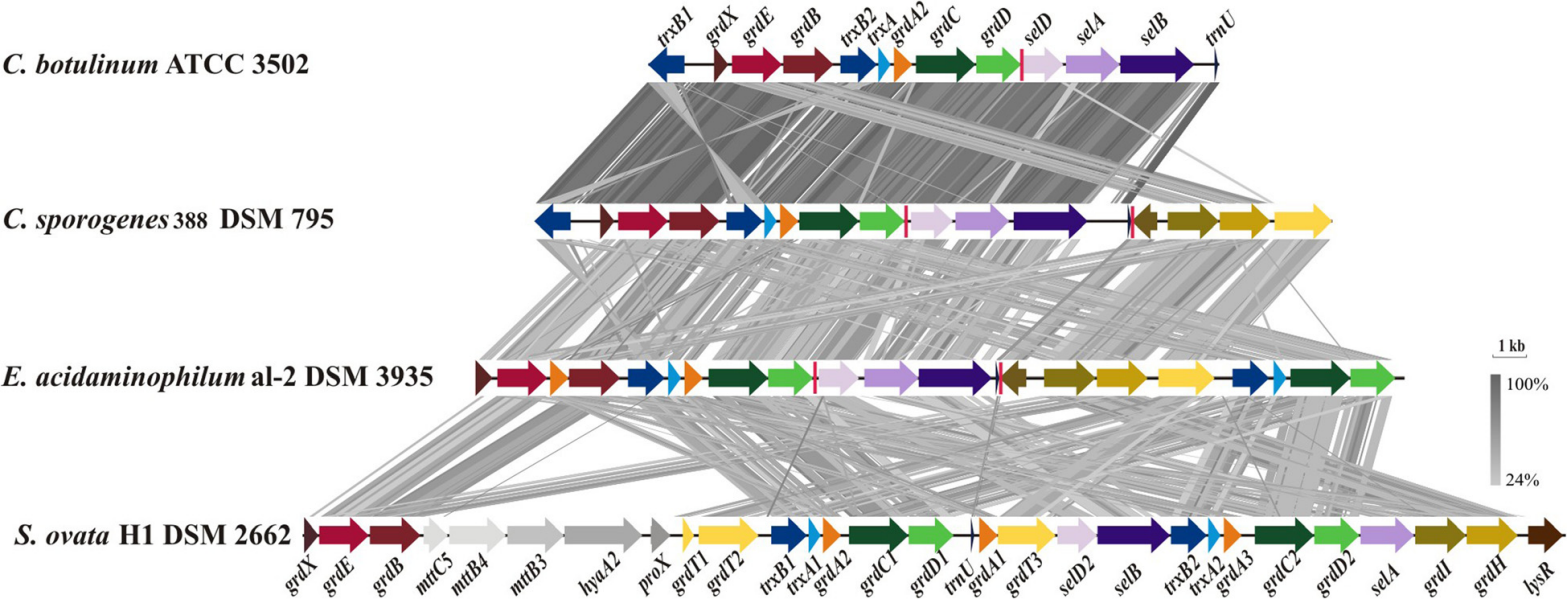
Tblastx comparison of glycine-, betaine-reductase and the *selABC* gene cluster of *C. sporogenes* DSM 795 with *C. botulinum* ATCC 3502, *E. acidaminophilum* al-2 DSM 3953 and *S. ovata* H1 DSM 2662: An E-value cutoff of 1e-10 was used and visualization was done with the program Easyfig [75]. The analyzed gene clusters are localized in different regions in the genomes of *C. sporogenes* DSM 795, *C. botulinum* ATCC 3502 as well as *E. acidaminophilum* al-2 DSM 3953. In *S. ovata* one genomic region includes all three gene clusters. Borders between the glycine-reductase (CSPO_4c08160-CSPO_4c08230), betaine-reductase (CSPO_4c10340-CSPO_4c10360) and *selABCtrnU* (CSPO_9c04980-CSPO_9c05010) gene cluster are indicated with red vertical lines.

9.42% of all protein coding genes were assigned to the COG category “amino acid transport and metabolism”, which indicates that utilization of amino acids plays an important role in the metabolism of *C. sporogenes*. Supporting this assumption, we identified several clusters coding for proteins involved in anaerobic amino acid degradation by the Stickland reaction [40-43]. One example is a gene cluster coding for several subunits of a proline reductase (CSPO_9c00030-CSPO_9c00180). This cluster contains two selenocysteine-containing proteins, the gamma subunit PrdB (CSPO_9c00070), and PrdC, a protein with high sequence homology to the C-subunit of Rnf-complex (CSPO_9c00100). The cluster shows a similar organization as in *C. sticklandii* [73,74], except for the additional presence of a transposase (CSPO_9c00150), a second copy of PrdH2 (CSPO_9c00160) and a non-selenocysteine-containing version of PrdC (CSPO_9c00170). Furthermore, we identified gene clusters for the degradation of glycine, another one for its derivative betaine (N,N,N-trimethylglycine), and a cluster involved in selenocysteine incorporation into proteins (Figure 5).

In contrast to *Sporomusa ovata* [76], in which all above mentioned gene are organized in one large cluster, the clusters identified in the genomes of *C. sporogenes*, *C.botulinum*ATCC 3502 and *Eubacterium acidaminophilum* al-2 DSM 3953 are localized in separate regions of the genomes. This is also true for the selenocysteine-incorporation genes (*selABC* [lilac tones]) as well as the Sec-specific tRNA (*trnU* [dark lilac]) as shown in Figure 5. The gene clusters of *C. sporogenes* show identical organization to those identified in *C. botulinum* and show only slightly differences to those found in *E. acidaminophilum* [77], whereas genes coding for the betaine-specific reductase is missing in the genome of *C. botulinum*. The glycine reductase cluster of *C. sporogenes* lacks the second copy of *grdA* [orange] in comparison to *E. acidaminophilum*, but contains two paralogs for thioredoxin reductases of the thioredoxin system [blue tones]. These two paralogs could also be identified in the cluster found in *C. botulinum*. In *C. sporogenes* the gene cluster coding for the betaine reductase (*grdRIH* [brown tones]) is much shorter than *E. acidaminophilum**’s* cluster, as genes coding for thioredoxin (*trxA* [light blue]) and thioredoxin reductase (*trxB* [dark blue]) as well as the two genes coding for the C-subunit of the reductase (*grdCD* [green tones]) are not present. The 32.5 kb comprising cluster of *S. ovata* includes genes coding for the glycine-specific subunit (*grdEB* [red tones]), genes coding for the betaine-specific subunit (*grdIH* [brown tones]), two copies of genes coding for the substrate-unspecific subunit C (*grdCD* [green tones]), and two copies coding for the thioredoxin and a thioredoxin reductase (*trxAB* [blue tones]). All these genes show identical clustering as identified in the genome of *C. sporogenes*. The genes coding for proteins necessary for the selenocysteine-incorporation show a different arrangement as identified in *C. sporogenes**,**C. botulinum* and *E. acidaminophilum*, where these genes are organized in a *selABCtrnU* operon [lilac tones] [78]. It is also visible in Figure 5 that genes coding for glycine-specific subunit (*grdBE* [red tones]) show high sequence homology to genes coding for betaine-specific subunit (*grdIH* brown tones]).

*C. sporogenes* is able to produce solvents such as ethanol and butanol [79,80]. The genome of *C. sporogenes*DSM 795 harbors the complete set of genes necessary for glycolysis (phosphoglucomutase, glucose-6-phophate isomerase, 6-phopsphofructokinase, 1-phosphofructokinase, fructose-bisphosphate aldolase, glyceraldehyde-3-phosphate dehydrogenase, aldehyde:ferredoxin oxidoreductase, glyceraldehyde-3-phosphate dehydrogenase, phosphoglycerate kinase, phosphoglycerate mutase, enolase, pyruvate kinase, pyruvate dehydrogenase) as well as aldehyde dehydrogenase and several bifunctional aldehyde-alcohol dehydrogenases, essential for ethanol production. Genes coding for key enzymes of butanol fermentation, such as butyryl-CoA dehydrogenase, acetyl-CoA acetyltransferase, 3-hydroxybutyryl-CoA dehydrogenase, 3-hydroxybutyryl-CoA dehydratase, several alcohol dehydrogenases, acetate kinase, phosphate acetyltransferase, two copies of butyrate kinase, two copies of phosphate butyryltransferase as well as three copies of formate acetyltransferase are also present (Additional file 2: Table S2). In contrast to other solventogenic clostridia, such as *C. beijerinckii*, *C. saccharobutylicum*, *C. saccharoperbutylacetonicum**,* or *C. acetobutylicum*, *C. sporogenes* is not able to produce acetone. In solventogenic clostridia, CoA transferase and acetoacetate decarboxylase, key enzymes of acetone production, are organized in the *sol* operon [81-86]. In *C. beijerinckii*, *C. saccharobutylicum*, and *C. saccharoperbutylacetonicum* aldehyde dehydrogenase is also part of this operon, whereas in *C. acetobutylicum* this enzyme is replaced by alcohol/aldehyde dehydrogenase. We could not identify CoA transferase and acetoacetate decarboxylase in the genome of *C. sporogenes*DSM 795 and both, alcohol/aldehyde dehydrogenase and aldehyde dehydrogenase are present, but located in different regions of the genome.

### Genome comparison

**Figure 6:**
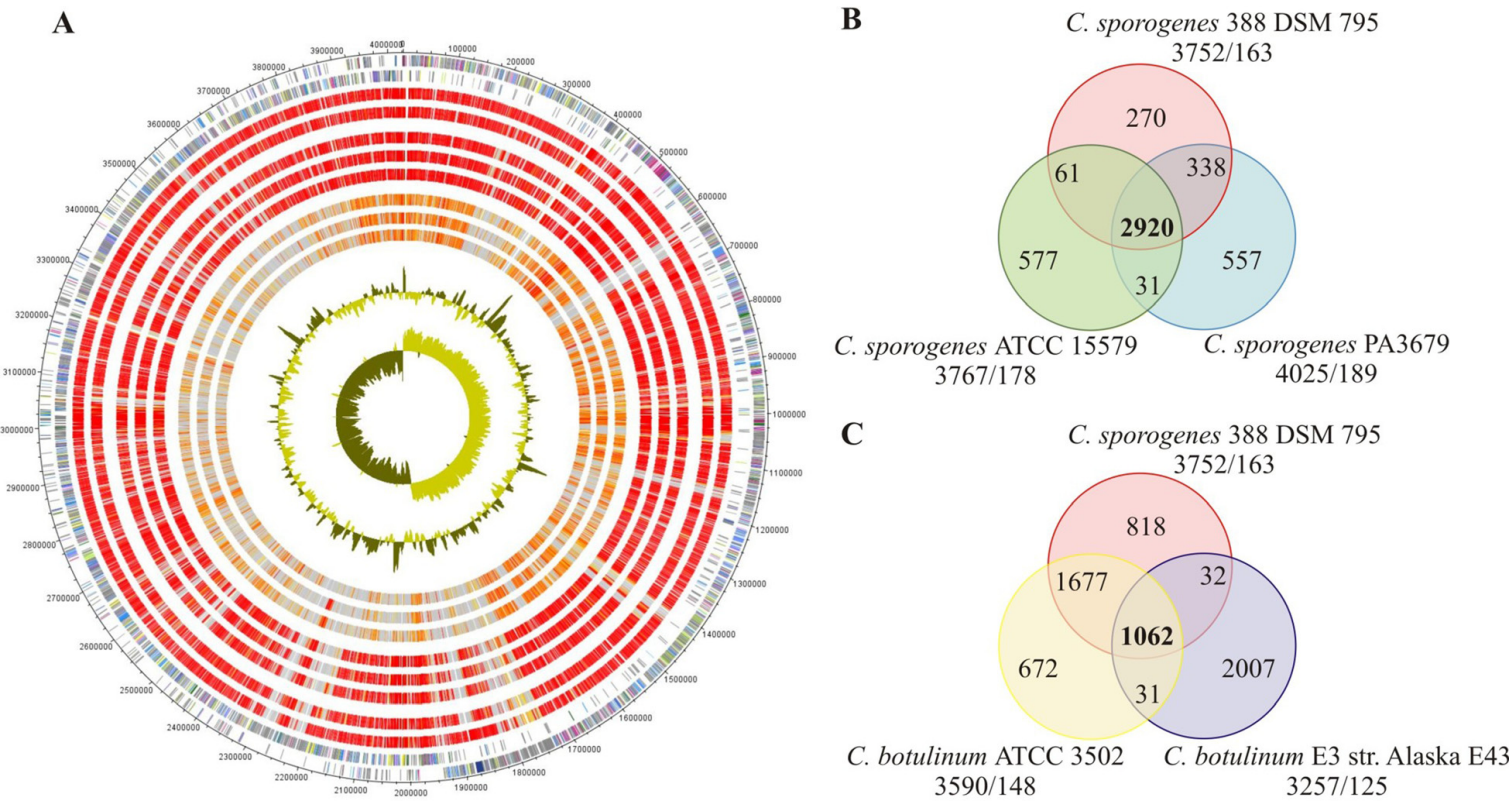
Genome comparison of *C. sporogenes* with different *C. sporogenes* and *C. botulinum* strains: **A**: Genes encoded by the leading and the lagging strand (circle 1 and 2) of *C. sporogenes* DSM 795 are marked in COG colors in the artificial chromosome map. The presence of orthologs (circle 3 to 10) is indicated for the genomes of *C. sporogenes* PA3679 (AGAH00000000), *C. sporogenes* ATCC 15579 (ABKW00000000) , *C. botulinum* ATCC 3502 (CP000727.1), *C. botulinum* B1 str. Okra (CP000939.1, CP000940.1), *C. botulinum* F str. Langeland (CP000728.1, CP000729.1), *C. botulinum* E3 str. Alaska E43 (CP001078.1), *C. botulinum* D str. 1873 (ACSJ01000001), *C. botulinum* C str. Eklund (ABDQ01000001) are illustrated in red to light yellow and singletons in grey (grey: >e^−10^-1; light yellow: <e^−50^- > e^−10^; gold: <e^−50^- > e^−90^; light orange: <e^−90^- > e^−100^; orange: <e^−100^- > e^−120^; red: <e^−120^-0). The two innermost plots represent the GC-content and the GC-skew. The artificial chromosome was built after scaffolding with Mauve alignment tool and concatenating the 16 contigs of the draft genome. Venn diagrams showing orthologs genes between the three sequenced *C. sporogenes* species **(B)** and between *C. sporogenes* DSM 795, the phylogenetic closely related *C. botulinum* ATCC 3502 and distantly related *C. botulinum* E3 str. Alaska E43 **(C)**. Ortholog detection was done with the Proteinortho software (blastp) with an identity cutoff of 50% and an E-value of 1e-10. The total number of genes and paralogs, respectively, were depicted under the corresponding species name.

*C. sporogenes* is renowned as a nontoxic surrogate for the proteolytic *C. botulinum**,* an organism which produces the botulinum neurotoxin (BoNT). *C. botulinum* is classified into seven serotypes (A to G) according to the neurotoxin antigenic specificity [57,87]. Serotypes A, B, E, and F cause human botulism, C and D are mainly described in animal toxicity, and no botulism case has been reported for serotype G [88,89]. For genome comparisons, two *C. sporogenes* species (ATCC 15579 and PA3679) and available representatives of all serotypes of *C. botulinum**,* except for serotype G*,* were chosen and retrieved from NCBI (Figure 6A). For this purpose and to prepare data for comparisons we used the scripts ncbi_ftp_download v0.2, cat_seq v0.1 and cds_extractor v0.6 [90]. Proteinortho v5.04 [91] was utilized to identify orthologs between the different organisms with an identity cutoff of 50% and an E-value of 1e-10. The core genome of all three *C. sporogenes* species consists of 2,920 CDS with a total pan genome of 4,754 CDS. *C. sporogenes*DSM 795 has 3,258 orthologous genes with *C. sporogenes* PA379 and 2,981 with *C. sporogenes*ATCC 15579 (Figure 6B). *C. sporogenes*DSM 795 has the least number of genome specific proteins of the three *C. sporogenes* strains with 270 singletons, as *C. sporogenes*ATCC 15579 contains 577 singletons and *C. sporogenes* PA3679 557. We identified 163, 178, and 189 paralogs in *C. sporogenes*DSM 795, ATCC 15579, and PA3679, respectively; but these genes were not included into analysis.

Phylogenetic analysis based on 16S rDNA revealed that *C. sporogenes* is closely related to serotypes A, B, and F *C. botulinum* strains, whereas it is distantly related to serotypes C, D, E, and G. These results were confirmed by gene content analyses as we identified 2,739 orthologous proteins between *C. sporogenes*DSM 795 and *C. botulinum*ATCC 3502 (serotype A) (Figure 6C). In contrast, there are only 1,094 orthologous genes between *C. sporogenes* and *C. botulinum* E3 str. Alaska E43 (serotype E). This number is nearly identical to the quantity of orthologs (1,093) found between *C. botulinum*ATCC 3502 and *C. botulinum* E3 str. Alaska E43. We identified 163, 148, and 125 paralogs in *C. sporogenes*DSM 795, *C. botulinum*ATCC 3502, and *C. botulinum* E3 str. Alaska E43, respectively; but these genes were not included into analysis.

**Figure 7:**
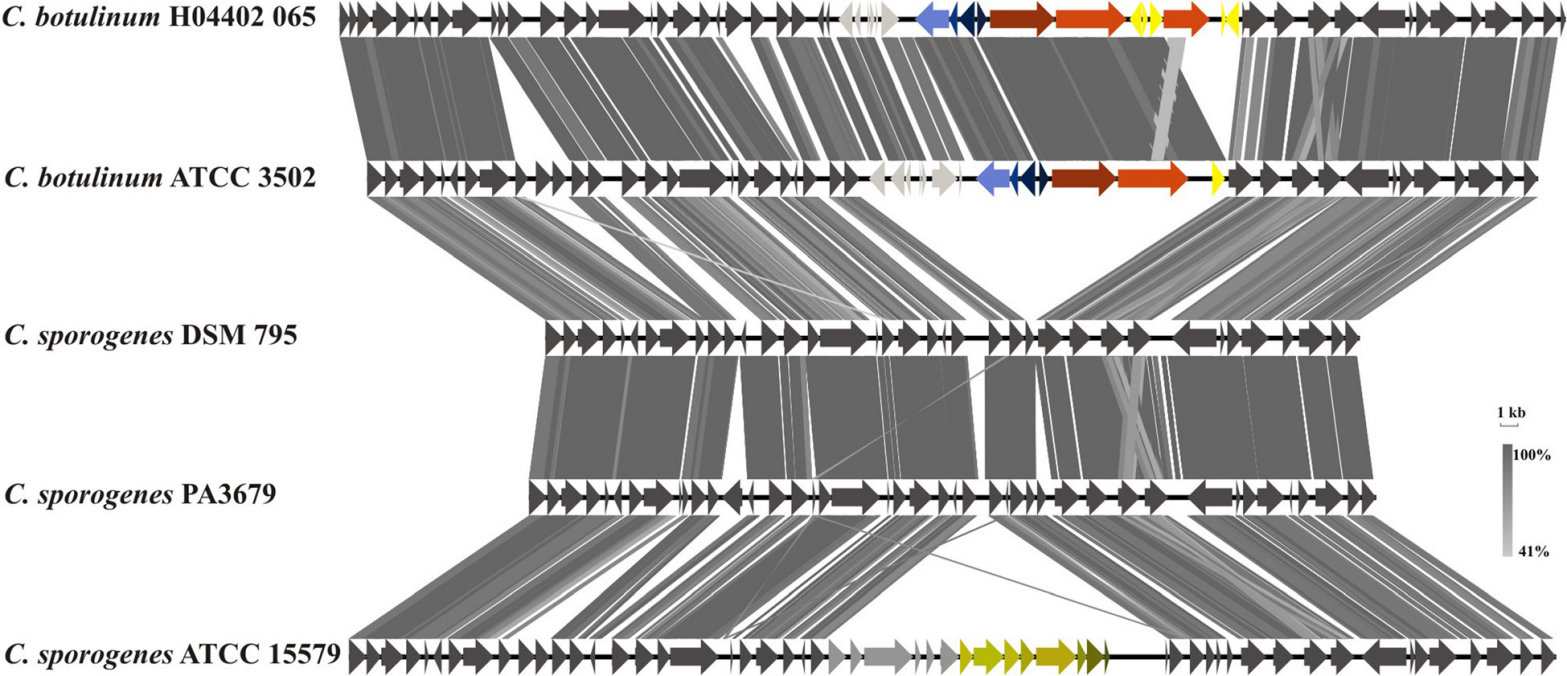
Tblastx comparison of the botulinum neurotoxin cluster (BoNT/A) and the flanking regions between *C. botulinum* and *C. sporogenes* strains. For the tblastx comparison an E-value cutoff of 1e-10 was set. Visualization was done with Easyfig. The neurotoxin cluster was marked in red tones, haemagglutinin components of the neurotoxin complex in blue tones, transposases in bright yellow, genes identified only in the *C. botulinum* strains in light grey, the CRISPR/cas system of *C. sporogenes* ATCC 15579 in olive tones, and singletons identified for the latter strain in grey. Core genes are anthracite-colored.

A genome comparison between *C. sporogenes*DSM 795 and *C. botulinum*ATCC 3502 revealed that both organisms have 2,739 orthologs in common, with 818 singletons in *C. sporogenes*DSM 795 and 672 singletons in *C. botulinum*ATCC 3502. The most important difference between both strains is the presence of the botulinum neurotoxin (BoNT/A) gene cluster in *C. botulinum*ATCC 3502 and its absence in *C. sporogenes*DSM 795 (Figure 7).

The region of the neurotoxin gene cluster is flanked by genes coding for several hypothetical proteins, components of different ABC transporters, as well as a ferrous iron transport system and several regulatory proteins (data not shown). As shown in Figure 7, these flanking genes are present in the *C. botulinum* strains as well as in all *C. sporogenes* strains. This region might be an area of high genome plasticity, as in *C. sporogenes*ATCC 15579 a subtype I-B/TNEAP CRISPR/cas system [92] is inserted, which could not be identified in the other strains used for this comparative approach.

## Conclusions

Members of the non-toxic species *C. sporogenes* are closely related to neurotoxin producer *C. botulinum*. This study presents an overview of physiological, morphological, and genomic characteristics of the type strain *C. sporogenes*DSM 795. Detailed insight into its proteolytic metabolism was gained on genomic level. Also, the ability of *C. sporogenes* to produce solvents such as ethanol and butanol was linked to a set of genes and compared to other solventogenic clostridia. Genome comparison of *C. sporogenes*DSM 795 with two other sequenced strains of this species revealed high similarity. *C. sporogenes*DSM 795 was also compared at the genomic level with two strains of the close relative *C. botulinum*.

## Additional files

Additional file 1: Table S1. Overview of all *C. botulinum* strains mentioned in this study [56]. Additional file 2: Table S2. Overview of enzymes, gene tags and locus tags of *C. sporogenes* DSM 795.
