# Supplementary material for: Genome sequence of Clostridium sporogenes DSM 795T, an amino acid-degrading, nontoxic surrogate of neurotoxin-producing Clostridium botulinum
Source: Stand Genomic Sci. 2015 Jul 21;10:40. doi: 10.1186/s40793-015-0016-y (PMC4517662; doi:10.1186/s40793-015-0016-y)
Supplement: Additional file 1: Table S1. — Overview of all C. botulinum strains mentioned in this study [56]. [file 40793_2015_16_MOESM1_ESM.docx]

**Table S1.** Overview of all [*C. botulinum*](http://dx.doi.org/10.1601/nm.3901) strains mentioned in this study [56]

| **Strain** | **Group** | **Serotype** | **GenBank/RefSeq ID** |
| --- | --- | --- | --- |
| Hall ([ATCC 3502](http://doi.org/10.1601/strainfinder?urlappend=%3Fid%3DATCC+3502)) | I | A1 | [CP000727](http://www.ncbi.nlm.nih.gov/nuccore/CP000727).1 |
| Kyoto | I | A2 | [CP001581](http://www.ncbi.nlm.nih.gov/nuccore/CP001581).1 |
| [H04402](http://www.ncbi.nlm.nih.gov/nuccore/H04402) 065 | I | A5 | [NC_017299](http://www.ncbi.nlm.nih.gov/nuccore/NC_017299) |
| Okra | I | B1 | [CP000939](http://www.ncbi.nlm.nih.gov/nuccore/CP000939).1, [CP000940](http://www.ncbi.nlm.nih.gov/nuccore/CP000940).1 |
| NCTC 7273 | I | B1 | - |
| Eklund | III | C | [ABDQ01000001](http://www.ncbi.nlm.nih.gov/nuccore/ABDQ01000001) |
| Pasteur 468 | III | C | - |
| 1873 | III | D | [ACSJ01000001](http://www.ncbi.nlm.nih.gov/nuccore/ACSJ01000001) |
| [ATCC 17851](http://doi.org/10.1601/strainfinder?urlappend=%3Fid%3DATCC+17851) | III | D | - |
| [ATCC 9564](http://doi.org/10.1601/strainfinder?urlappend=%3Fid%3DATCC+9564) | II | E1 | - |
| Alaska E43 | II | E3 | [CP001078](http://www.ncbi.nlm.nih.gov/nuccore/CP001078).1 |
| ATCC 25764 | I | F | - |
| Langeland | I | F | [CP000728](http://www.ncbi.nlm.nih.gov/nuccore/CP000728).1, [CP000729](http://www.ncbi.nlm.nih.gov/nuccore/CP000729).1 |
| 113/30 | IV | G | - |
