# Supplementary material for: Genome sequence of Clostridium sporogenes DSM 795T, an amino acid-degrading, nontoxic surrogate of neurotoxin-producing Clostridium botulinum
Source: Stand Genomic Sci. 2015 Jul 21;10:40. doi: 10.1186/s40793-015-0016-y (PMC4517662; doi:10.1186/s40793-015-0016-y)
Supplement: Additional file 2: Table S2. — Overview of enzymes, gene tags and locus tags of C. sporogenes DSM 795. [file 40793_2015_16_MOESM2_ESM.docx]

**Table S2.** Overview of enzymes, gene tags, and locus tags of [*C. sporogenes*](http://dx.doi.org/10.1601/nm.4021) [DSM 795](http://doi.org/10.1601/strainfinder?urlappend=%3Fid%3DDSM+795)

| **Function** | **Gene Name** | **IDs** |
| --- | --- | --- |
| phosphoglucomutase | *pgcA* | CSPO_9c06430 |
| glucose-6-phosphate isomerase | *pgi* | CSPO_1100310 |
| 6-phosphofructokinase | *pfkA* | CSPO_11c01590 |
| 1-phosphofructokinase | *fruK* | CSPO_6c01400 |
| fructose-bisphosphate aldolase | *fba* | CSPO_1c02230, |
| glyceraldehyde-3-phosphate dehydrogenase | *gap* | CSPO_4c06570 CSPO_1c01910 |
| aldehyde:ferredoxin oxidoreductase | *aor* | CSPO_6c00760 |
| glyceraldehyde-3-phosphate dehydrogenase | *gapN* | CSPO_4c08140 |
| phosphoglycerate kinase | *pgk* | CSPO_1c01900 |
| phosphoglycerate mutase | *gpmI* | CSPO_1c01880 |
| enolase | *eno* | CSPO_1c01870 |
| pyruvate kinase | *pyk* | CSPO_11c01580 |
| pyruvate dehydrogenase | *pdh* | CSPO_4c12440-CSPO_4c12480 |
| aldehyde dehydrogenase | *ald* | CSPO_4c06610 |
| alcohol/aldehyde dehydrogenase | *adhE* | CSPO_1c00730, CSPO_6c02620, CSPO_6c027302 |
| butyryl-CoA dehydrogenase | *bcd* | CSPO_10c00770 |
| acetyl-CoA acetyltransferase | *thlA* | CSPO_10c00780 |
| 3-hydroxybutyryl-CoA dehydrogenase | *hbd* | CSPO_10c00790 |
| 3-hydroxybutyryl-CoA dehydratase | *crt* | CSPO_10c00800 |
| alcohol dehydrogenase | *bdhA* | CSPO_4c10040 |
| alcohol dehydrogenase | *adh* | CSPO_4c12770, CSPO_6c02790 |
| acetate kinase | *ackA* | CSPO_8c00990 |
| phosphate acetyltransferase | *pta* | CSPO_8c00100 |
| butyrate kinase | *buk* | CSPO_11c02180, CSPO_11c02200 |
| phosphate butyryltransferase | *ptb* | CSPO_9c06470, CSPO_11c02190 |
| formate acetyltransferase | *pfl* | CSPO_6c02720, CSPO10c00610, CSPO_10c00930 |
